# Sudden onset of sleep caused by hypothalamic infarction: a case report

**DOI:** 10.1186/s12883-019-1414-3

**Published:** 2019-08-02

**Authors:** Takeo Matsubara, Keisuke Suzuki, Akiko Kawasaki, Masayuki Miyamoto, Madoka Okamura, Takashi Kanbayashi, Hidehiro Takekawa, Toshiki Nakamura, Yuji Watanabe, Masanori Matsubara, Koichi Hirata

**Affiliations:** 1 Department of Neurology, Dokkyo Medical University, 880 Kitakobayashi, Mibu, Shimotsuga, Tochigi, 321-0293 Japan; 2 Department of Clinical Medicine for Nursing, Dokkyo Medical University School of Nursing, Tochigi, Japan; 3 International Institute for Integrative Sleep Medicine (WPI-IIIS), University of Tsukuba, Tsukuba, Japan; 4 Center of Medical Ultrasonics, Dokkyo Medical University Hospital, Tochigi, Japan; 5 Stroke Center, Dokkyo Medical University Hospital, Tochigi, Japan; 6 Department of Neurology, Rehabilitation Amakusa Hospital, Saitama, Japan

**Keywords:** Hypothalamic infarction, Sudden onset of sleep, Hypersomnia, Horner syndrome

## Abstract

**Background:**

Hypothalamic lesions, such as tumors and demyelinating diseases, reportedly cause abnormal sleepiness. However, stroke involving the hypothalamus has rarely been described. Here, we report a patient with infarction restricted to the hypothalamus who presented with sudden onset of sleep.

**Case presentation:**

A 42-year-old woman with a history of migraine without aura presented with irresistible sleepiness and developed several episodes of sudden onset of sleep. Neurological examinations were unremarkable except for partial left Horner syndrome. Brain magnetic resonance imaging (MRI) revealed a high-intensity lesion restricted to the left hypothalamus on diffusion-weighted and fluid-attenuated inversion recovery MRI images. Cerebrospinal fluid (CSF) orexin-A levels obtained on hospital day 3 after her sleepiness had resolved were normal (337 pg/mL; normal > 200 pg/mL). Serum anti-nuclear and anti-aquaporin 4 (AQP4) antibodies and CSF myelin basic protein and oligoclonal band were negative. A small hypothalamic infarction was suspected, and the patient was treated with intravenous edaravone and argatroban, as well as oral clopidogrel. Three months later, there had been no clinical relapse, and the hypothalamic lesion had almost disappeared on follow-up MRI. No new lesion suggestive of demyelinating disease or tumor was observed.

**Conclusion:**

Hypothalamic stroke should be considered a cause of sudden onset of sleep.

## Background

The hypothalamus plays a key role in regulating sleep, wakefulness, temperature, and food intake. Hypothalamic lesions due to tumor and demyelinating diseases (e.g., multiple sclerosis and neuromyelitis optica spectrum disorders) reportedly cause abnormal sleepiness, and reduced orexin-A levels in the cerebrospinal fluid (CSF) have been observed in those patients, suggesting that an impaired orexinergic system participates in the development of hypersomnia [1]. We recently reported decreased CSF orexin levels in 3 of 4 central nervous system lupus patients with hypothalamic lesions and excessive daytime sleepiness [2]. However, hypersomnia following hypothalamic infarction has rarely been documented [3, 4]. Herein, we present a patient with a history of migraine who developed several episodes of sudden onset of sleep due to a small infarct in the posterior medial part of the hypothalamus.

## Case presentation

**Fig. 1:**
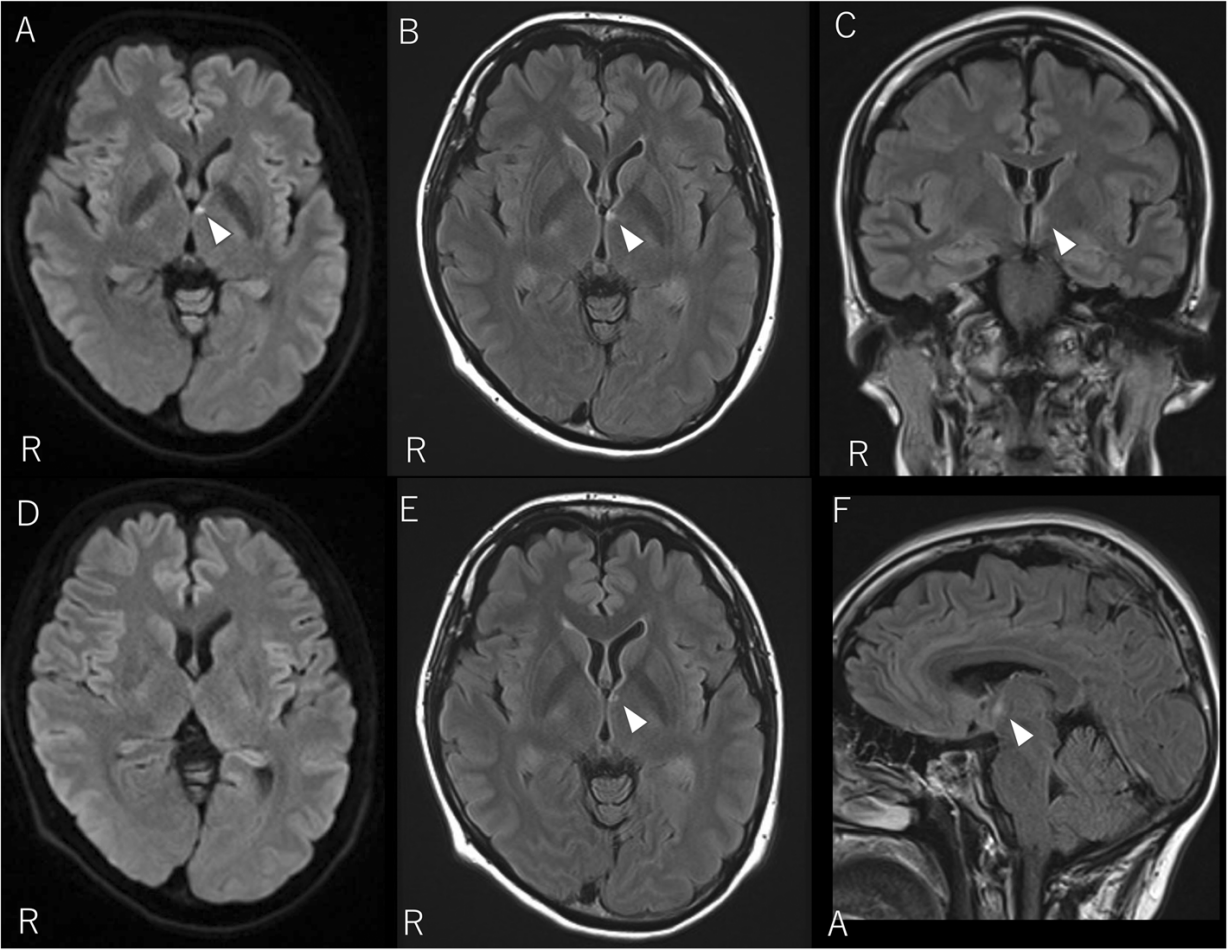
Brain MRI of the patient. Diffusion-weighted (**a**) and fluid-attenuated inversion recovery MRI images (**b**, **c**) obtained on admission reveal a small, high-signal intensity restricted to the left hypothalamus (arrowheads). Sagittal fluid-attenuated inversion recovery MRI shows involvement of the posterior part of the hypothalamus (**f**, arrowhead). Follow-up MRI after 3 months shows unremarkable findings on diffusion-weighted images (**d**) and reduced intensity of the hypothalamic lesion on fluid-attenuated inversion recovery MRI images (**e**) (arrowhead)

A 42-year-old woman noticed irresistible sleepiness around noon. On the same day, she developed sudden onset of sleep several times while standing as she visited her child’s classroom. Each episode lasted for a few seconds. Excessive daytime sleepiness persisted throughout that day but resolved on the following day. The patient had quit smoking 10 years ago. The patient’s sleep habits were regular; she slept 8 h/night from 11 PM to 7 AM. The patient had been treated for migraine without aura for 24 years and had a history of surgical treatment for an ovarian cyst. The patient was not on regular medicine but took loxoprofen for migraine attacks. At the initial presentation, the patient was alert. The pupil sizes were asymmetric (right 4 mm, left 2.5 mm). The pupillary reflexes to light were normal, and left-sided ptosis was noted. There was no disturbance of sweating. Facial sensations and facial muscles were intact. No motor weaknesses or sensory disturbances were noted. Diffusion-weighted (Fig. 1a) and fluid-attenuated inversion recovery magnetic resonance imaging (MRI) revealed a high-intensity lesion restricted to the posterior part of the left hypothalamus (Fig. 1b, c, f). Magnetic resonance angiography showed no stenosis or occlusion in the main cerebral arteries. Atherosclerotic changes were unremarkable on carotid artery sonography. An electrocardiogram showed normal tracing. On transthoracic and transesophageal echocardiography, a potential cardiac source of an embolism or the right-to-left shunt, including patent foramen ovale, was not detected. On laboratory exams, liver function, renal function, glucose, cholesterol, soluble interleukin-2 receptor, c-reactive protein, erythrocyte sedimentation rate, homocysteine, protein C, protein S, antithrombin III, prothrombin time, activated partial thromboplastin time and D-dimer levels (0.8 μg/ml) were normal. The levels of pituitary hormones, such as thyroid stimulating hormone, adrenocorticotropic hormone, follicle stimulating hormone, luteinizing hormone, growth hormone, and prolactin, were normal. Anti-nuclear antibody, anti-SS-A/B antibody, MPO-ANCA, and β-2-glycoprotein I were all negative. CSF analysis was normal (2 mononuclear cells/μL and a protein level of 33 mg/dL). CSF orexin-A levels obtained on hospital day 3 were normal (337 pg/mL; normal > 200 pg/mL). Serum anti-aquaporin 4 (AQP4) antibody, CSF myelin basic protein and oligoclonal band were negative. Electroencephalography showed a background of 10–11 Hz alpha rhythms without epileptic discharges. A small infarction restricted to the hypothalamus was suspected, and the patient was treated with intravenous edaravone and argatroban, as well as oral clopidogrel. She did not complain of recurrence of sleepiness after admission. Her eye symptoms spontaneously improved on hospital day 2. Three months later, there had been no clinical relapse, and follow-up MRI was performed. Diffusion-weighted imaging was unremarkable (Fig. 1d), and fluid-attenuated inversion recovery MRI showed reduced intensity of the hypothalamic lesion (Fig. 1e). There was no new lesion suggestive of demyelinating disease or tumor.

## Discussion and conclusions

Our patient exhibited several short episodes of sudden onset of sleep and partial Horner syndrome due to a small infarct in the unilateral hypothalamus. In our patient, left-sided ptosis was considered partial Horner syndrome rather than incomplete oculomotor nerve palsy because there was no limitation of extraocular movement and the ipsilateral pupil was smaller (2.5 mm) than the contralateral pupil (4 mm). Horner syndrome due to hypothalamic stroke has been previously reported [3, 5, 6]. Isolated hypothalamic infarction is rare because of the rich vascular supply and anastomosis of this region. In a study examining anastomoses among the hypothalamic arteries in 14 human brains, there were 5 to 22 channel-like anastomoses on either side of the brain. The anastomoses most often involved were the commissural arteries, superior hypophyseal arteries and the tuberoinfundibular branches of the posterior communicating artery [7]. We summarized the characteristics of 6 patients with hypothalamic stroke with or without hypersomnia, including our patient (Table 1) [3–6, 8]. The size of the hypothalamic lesion of our patient was as small as that of a patient reported by Smith et al. [8] but was significantly smaller than those of other patients [3–6]. Three of 6 (50%) patients exhibited hypersomnia, and CSF orexin levels were measured in 2 of those 3 patients. Scammell et al. [4] reported that a 23-year-old man developed hypersomnia and cataplexy following bilateral hypothalamic infarctions after removal of craniopharyngioma. Short sleep latency, sleep-onset rapid eye movement (REM) periods and reduced CSF orexin-A levels (167 pg/ml) led to a diagnosis of narcolepsy secondary to hypothalamic stroke. In our patient who presented with several episodes of sudden onset of sleep, the CSF orexin-A level was within the normal range (337 pg/ml). Moreover, the lesion on the MRI images was small and confined to the left medial to posterior hypothalamus in our patient. A possible reason for the normal CSF orexin-A level in our patient was that she had no sleepiness at the time of the CSF examination; therefore, it is possible that the posterolateral parts of the hypothalamus may have been more extensively involved at the initial onset. Additionally, arousal systems other than the orexinergic system, such as histaminergic tuberomammillary neurons, may have been involved. In a study including 6 patients with bilateral paramedian thalamic infarctions, CSF orexin-A levels were decreased in 2 patients with rostral midbrain involvement and were within normal ranges in 4 patients without midbrain involvement [9]. Hypersomnia in paramedian thalamic stroke is known as “pseudohypersomnia”, characterized by increased stage N1, decreased stage N2 and spindles during sleep; however, REM sleep is unaffected [10, 11].

**Table 1:** Characteristics of patients with hypothalamic stroke with and without hypersomnia

Patient no.	Present case	Bassetti et al. [5]	Austin et al. [3]	Stone et al. [6]	Smith et al. [8]	Scammell et al. [4]
Age (years)/Sex	42/F	36/M	62/F	67/M	40/M	23/M
Hypersomnia	+	–	+	–	–	+
Other clinical features	Left ptosis	Right Horner syndrome	Left-sided occipital headache, left-sided ptosis, and right-sided face and arm weakness	Right arm weakness, right Horner syndrome	Hyperhidrosis on the left face, arm, chest, abdomen, and leg	Gonadal hypoplasia and delayed puberty.
Habits	Quit smoking and drinking 10 years ago	Not available	Not available	Not available	No smoking	Not available
Comorbid diseases/past medical history	Migraine without aura	Not available	Diabetes mellitus, hypertension, coronary artery disease	Not available	(−)	A large craniopharyngioma was identified below his posterior hypothalamus and was surgically removed.
Hypothalamic lesions on MRI	The left posterolateral part of hypothalamus	The occipital lobe, inferomedial temporal lobe, anterolateral midbrain and ventroposterolateral thalamo-subthalamic area	The left posterior hypothalamus, the genu and posterior limb of the internal capsule.	The left hypothalamus and anterior thalamus	The right posterior hypothalamus	The bilateral, nearly entire, hypothalamus
CSF orexin-A levels (pg/ml)	337	Not available	Not available	Not available	Not available	167

The etiology of stroke in our patient is unclear. The patient had no vascular risk factors, such as hypertension, dyslipidemia or diabetes, and had quit smoking 10 years before. There were no atherosclerotic changes on carotid artery sonography, coagulation abnormalities or antibodies related to central nervous system vasculitis on laboratory testing. Large vessel vasculitis, such as Takayasu’s arteritis and temporal arteritis, were unlikely as inflammatory markers (c-reactive protein and erythrocyte sedimentation rate) were not elevated. The patient had migraine, but this event was not accompanied by a preceding aura, which is a risk factor for stroke among young individuals [12]. Patent foramen ovale was not found, and cardiac and aortic sources of embolism were unlikely based on transthoracic and transesophageal echocardiography. However, in our patient, artery-to-artery embolism, arterial dissection without headache, primary angiitis of the central nervous system or intravascular lymphoma with normal serum soluble interleukin-2 receptor could not be completely ruled out.

As a limitation, we did not perform polysomnography or multiple sleep latency tests to evaluate the patient’s sleep structure and abnormal sleepiness because abnormal sleepiness and sudden onset of sleep had resolved just before admission. Additionally, the CSF orexin measurement was performed after recovery from abnormal sleepiness.

In conclusion, we reported a patient with hypothalamic infarction who presented with sudden onset of sleep and hypersomnia. Thus, hypothalamic stroke should be considered a cause of sudden onset of sleep.

## Data Availability

All data collected by clinical examination, laboratory and imaging data, and a review of the patient’s medical record are presented in the manuscript.

## Abbreviations

CSF: Cerebrospinal fluid
MRI: Magnetic resonance imaging
REM: Rapid eye movement
